# Role of the S1P pathway and inhibition by fingolimod in preventing hemorrhagic transformation after stroke

**DOI:** 10.1038/s41598-019-44845-5

**Published:** 2019-06-05

**Authors:** Angélica Salas-Perdomo, Francesc Miró-Mur, Mattia Gallizioli, Vanessa H. Brait, Carles Justicia, Anja Meissner, Xabier Urra, Angel Chamorro, Anna M. Planas

**Affiliations:** 1 Departament d’Isquèmia Cerebral i Neurodegeneració, Institut d’Investigacions Biomèdiques de Barcelona (IIBB), Consejo Superior de Investigaciones Científicas (CSIC), Barcelona, Spain; 2 Àrea de Neurociències, Institut d’Investigacions Biomèdiques August Pi i Sunyer (IDIBAPS), Barcelona, Spain; 3 Functional Unit of Cerebrovascular Diseases, Hospital Clínic, Barcelona, Spain; 4 Present Address: The Florey Institute of Neuroscience and Mental Health, University of Melbourne, Parkville, Victoria Australia; 5 Present Address: Department of Experimental Medical Sciences & Wallenberg Center for Molecular Medicine, Lund University, Lund, Sweden

**Keywords:** Mouse, Stroke

## Abstract

Hemorrhagic transformation (HT) is a complication of severe ischemic stroke after revascularization. Patients with low platelet counts do not receive reperfusion therapies due to high risk of HT. The immunomodulatory drug fingolimod attenuated HT after tissue plasminogen activator in a thromboembolic stroke model, but the underlying mechanism is unknown. Fingolimod acts on several sphingosine-1-phosphate (S1P) receptors, prevents lymphocyte trafficking to inflamed tissues, and affects brain and vascular cells. This study aimed to investigate changes in S1P-signaling in response to brain ischemia/reperfusion and the effects of the S1P receptor modulator fingolimod on HT. We studied brain expression of S1P signaling components, S1P concentration, and immune cell infiltration after ischemia/reperfusion in mice. We administered fingolimod after ischemia to wild-type mice, lymphocyte-deficient Rag2^−/−^ mice, and mice with low platelet counts. Ischemia increased S1P-generating enzyme SphK1 mRNA, S1P concentration, and S1P receptor-1 (S1P1)^+^ T-cells in the brain. Fingolimod prevented lymphocyte infiltration, and attenuated the severity of HT in Rag2^−/−^ mice but it was ineffective under thrombocytopenia. Fingolimod prevented β-catenin degradation but not Evans blue extravasation. Ischemia/reperfusion upregulates brain S1P signaling pathway, and fingolimod exerts local effects that attenuate HT. Although fingolimod seems to act on the brain tissue, it did not prevent blood-brain barrier leakage.

## Introduction

Hemorrhagic transformation (HT) is a complication of severe ischemic stroke that mainly occurs after reperfusion therapies^1^. Activation of metalloproteinases and severe endothelial damage following ischemia/reperfusion compromise the endothelial integrity facilitating the development of hemorrhage^2–4^. Certain conditions, such as low platelet counts, increase the risk of bleeding and reperfusion therapies are not recommended in these ischemic stroke patients^5^. Likewise, prior antiplatelet therapy is independently associated with HT after thrombolysis^6^. Treatments able to increase the safety of reperfusion therapies might benefit patients that are currently excluded from these treatments due to a high risk of HT. Fingolimod attenuates HT after brain ischemia in mice that received tissue plasminogen activator (tPA)^7^, and preserves the blood-brain barrier (BBB) in a model of experimental autoimmune encephalomyelitis^8^. Fingolimod has protective effects in experimental ischemic stroke by reducing infarct volume and ameliorating neurological deficits^9^. Two small clinical trials showed that fingolimod reduced infarct growth and improved neurological deficits in patients with ischemic stroke^10,11^. In combination with tPA, fingolimod also reduced microvascular permeability and the risk of hemorrhagic complications in ischemic stroke patients. A clinical trial is ongoing to assess whether the combination of fingolimod with reperfusion therapies is effective in attenuating reperfusion injury in patients with large vessel occlusion treated with 6 h of stroke onset^12^.

Fingolimod is an immunomodulatory drug binding the sphingosine-1-phosphate (S1P) receptors 1, 3, 4 and 5^13^. S1P, a bioactive phospholipid with a complex metabolism, has recently gained more attention due to its association with the development of several cardiovascular and inflammatory diseases. Two distinct kinases (SphK1 and SphK2) generate S1P from sphingosine in response to various stimuli, including inflammatory cues^14^. SphK1 can be activated by several conditions, including inflammatory stimuli, locally upregulating S1P production^14^. Due to the pleiotropic effects of S1P, tight regulation of S1P level is vital for the control of several important cellular functions, including the regulation of S1P gradients involved in immune cell trafficking^15^. S1P-governed chemotaxis is mediated by specific S1P receptor expression on immune cells: T-cells for instance, require mostly S1P receptor type 1 (S1P1) to migrate along the S1P gradient^16^. This has been successfully exploited for clinical applications^13^. Fingolimod is a prodrug that becomes biologically active after phosphorylation, which *in vivo* appears to be mainly driven by SphK2^17^. Fingolimod causes severe lymphopenia through its action on S1P receptor 1 (S1P1) by blocking the egress of lymphocytes from secondary lymphoid organs and preventing them from reaching inflamed tissues^18,19^. Due to the deleterious effects of T cells in the acute phase of ischemic stroke^20,21^, blockade of T cell migration to the brain is recognized as an important mechanism underlying the protective effects of fingolimod in mice after ischemia/reperfusion. This concept is supported by the failure of fingolimod to reduce the infarct volume in lymphocyte-deficient mice^22^. Furthermore, a selective S1P1 agonist reduced infarct volume after ischemia/reperfusion in mice only at doses able to induce sustained lymphopenia^23^. However, fingolimod crosses the BBB and, beyond the effects on immune cells, it exerts multiple actions in brain and vascular cells^24–26^ due to the wide expression of S1P receptors in different cell types, including endothelial cells^27,28^.

This study aimed to investigate the effects of brain ischemia/reperfusion on the S1P pathway and the action of fingolimod. To this end we studied ischemia-induced changes in the expression of S1P signaling components in the brain, cerebral S1P concentration, and leukocyte infiltration to the ischemic brain tissue. Moreover, we focused on the effects of fingolimod on ischemia-induced HT, including conditions favoring HT such as relative thrombocytopenia.

## Results

### Ischemia upregulates the cerebral S1P pathway

**Figure 1:**
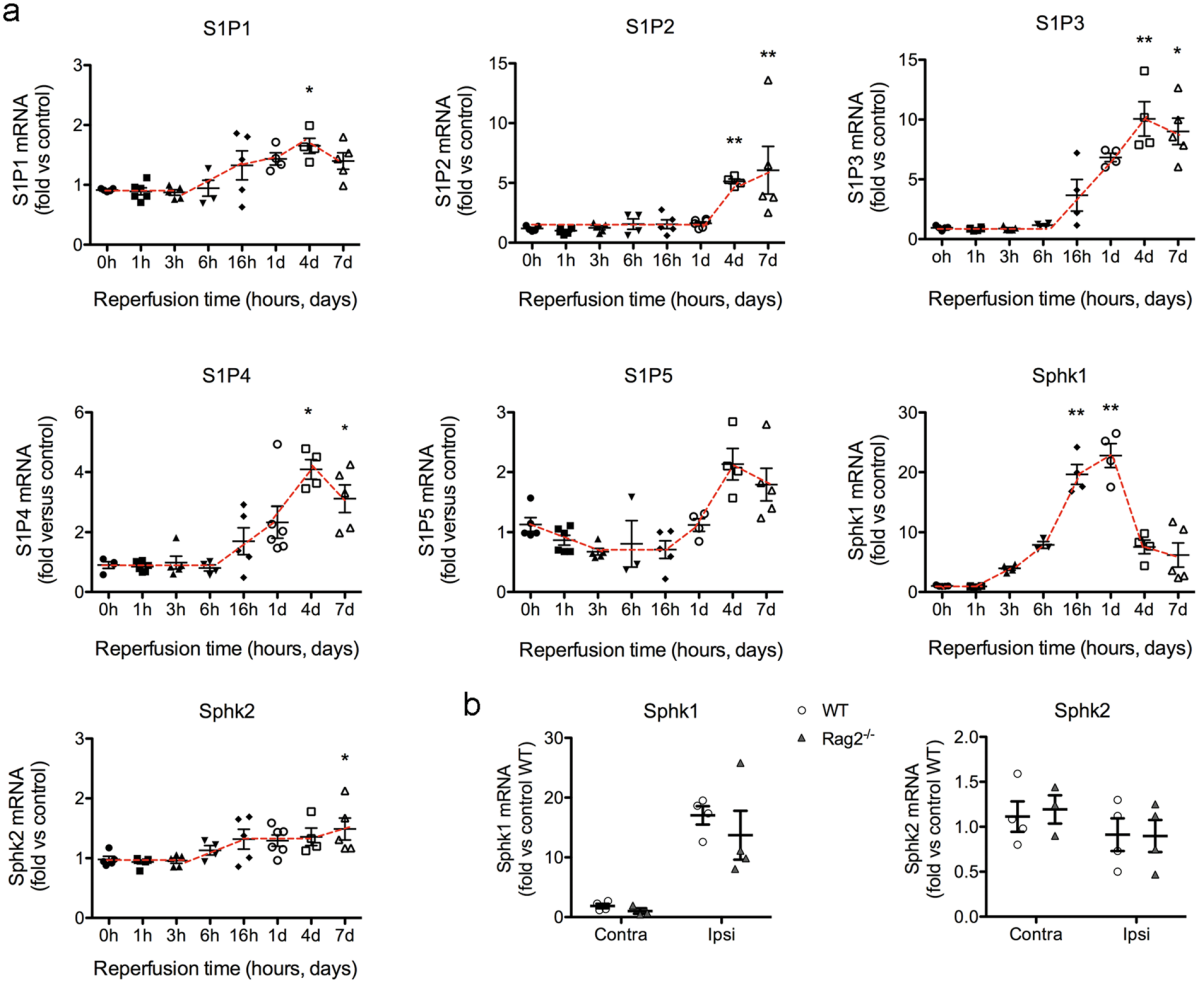
Time-dependent changes in mRNA expression of genes involved in the S1P signaling axis. (**a**) Mice (n = 41) we subjected to 45 min MCAo and were sacrificed at different time points: immediately without reperfusion (time 0) (n = 6) or at 1 h (n = 6), 3 h (n = 5), 6 h (n = 4), 16 h (n = 5), 24 h (n = 6), 4 days (n = 4), or 7 days (n = 5) after reperfusion. mRNA expression of the genes encoding for S1P receptors S1P1, S1P2, S1P3, S1P4 and S1P5, and for the kinases generating S1P, Sphk1 and Sphk2 in the ipsilateral (ischemic) hemisphere were assessed. Results are expressed as fold versus non-ischemic control brain tissue. S1P1, S1P3 and S1P4 mRNAs increased from 16 h of reperfusion, peaking after 4 days. S1P2 mRNA did not changed until day 4 and 7, whereas increases in S1P5 mRNA were not statistically significant. Sphk1 mRNA was strongly upregulated, with increases already detected at 3 h of reperfusion, peaking at 24 h, and then declining. In contrast, the mRNA expression of Sphk2 was rather stable and only showed a small tendency to increase progressively and reached statistically significant differences versus control at day 7. Kruskal-Wallis test followed by Dunn’s test. *p < 0.05, **p < 0.01 vs. time 0. (**b**) The expression of Sphk1 and Sphk2 mRNA was studied 24 h post-ischemia in the ipsilateral (Ipsi) and contralateral (Contra) hemispheres of an independent group of wild type mice (WT) and Rag2^−/−^ mice 24 h post-ischemia (n = 4 per group). The increase in Sphk1 mRNA induced by ischemia did not differ between genotypes (Two-way ANOVA, genotype effect p = 0.402, hemisphere effect p < 0.001, interaction p = 0.614). SphK2 mRNA expression did not change between genotypes. Values are expressed as fold versus wild type control.

We studied the time course of mRNA expression of genes of the S1P pathway, including the S1P receptors (S1P1, S1P2, S1P3, S1P4 and S1P5) and the kinases involved in S1P generation, Sphk1 and Sphk2 (Fig. 1). Ischemia induced by 45-min intraluminal occlusion of the middle cerebral artery (MCAo) induced significant increases in S1P receptor mRNA expression (Fig. 1a) that peaked around 4 days post-ischemia. These delayed increases indicate the possible association of S1P receptors with the glial and endothelial reactions or infiltrating leukocytes. The S1P receptor mRNA showing the highest up-regulation was S1P3. The expression of Sphk2 mRNA showed a progressive weak increase that became statistically significant 7 days post-ischemia. In contrast, expression of Sphk1 mRNA showed a prominent increase after ischemia that was detected as early as 3 h after reperfusion, peaking at 24 h, and declining afterwards (Fig. 1a). We verified that the ischemia-induced increase in Sphk1 mRNA was independent of lymphocytes since it also occurred in lymphopenic Rag2^−/−^ mice 24 h post-ischemia (Fig. 1b). Likewise, the expression of Sphk2 was similar in Rag2^−/−^ mice and wild type mice at this time point (Fig. 1b).

**Figure 2:**
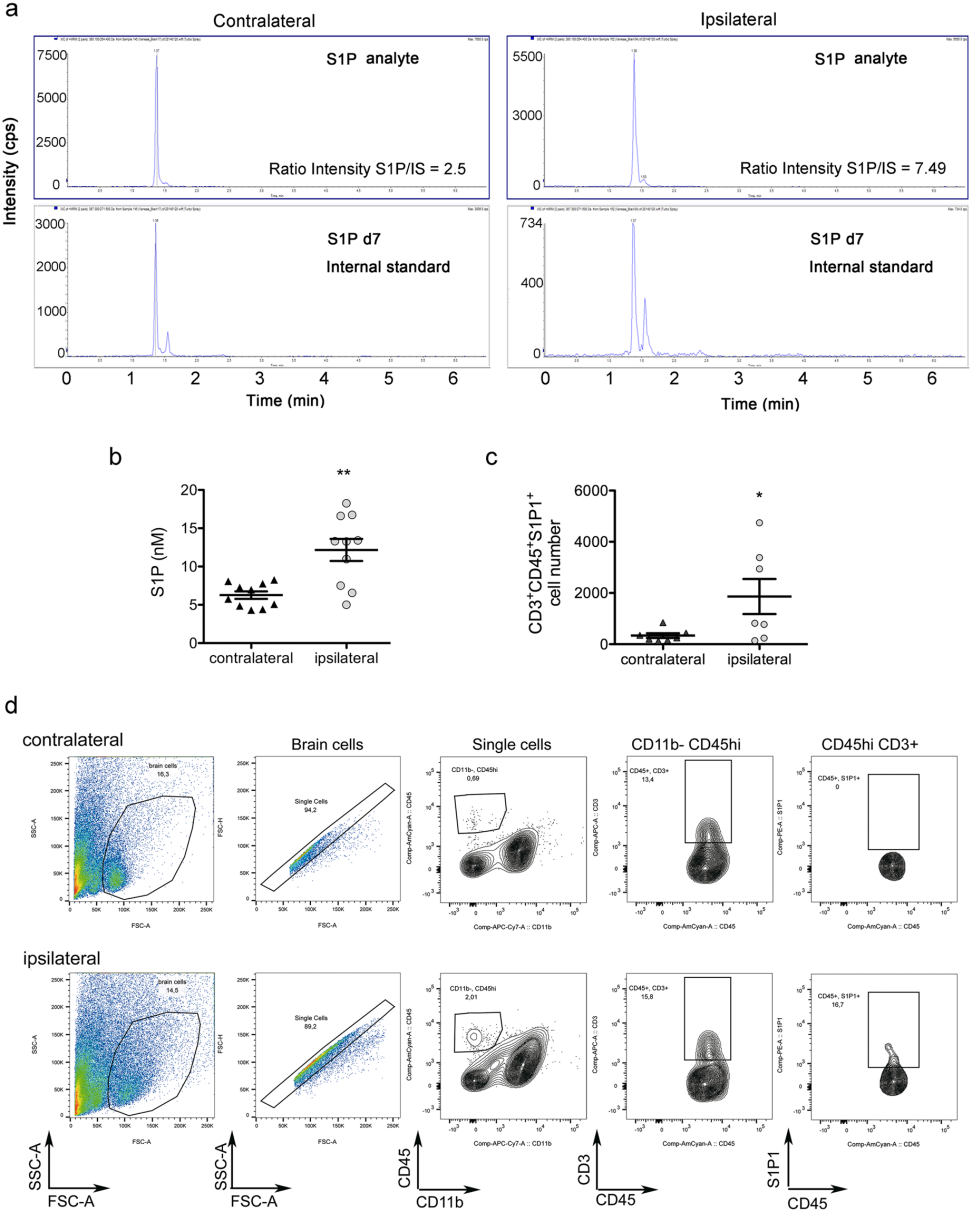
Ischemia increases the brain concentration of S1P and the recruitment of S1P^+^ T cells. (**a,b**) S1P concentration was measured by mass spectrometry in the contralateral and ipsilateral (ischemic) brain hemispheres 24 h after MCAo (n = 10 mice). Illustrative ionograms of representative samples of each brain hemisphere and the corresponding internal standard (IS) are shown in (**a**). Cps indicates counts per second. Ischemia significantly increased S1P concentration in the ipsilateral hemisphere (Mann-Whitney test, **p = 0.007) (**b**). (**c**) The presence of S1P1^+^ T cells in the ipsilateral and contralateral brain hemispheres was evaluated 4 days post-ischemia by flow cytometry (n = 7). Ischemia significantly increased S1P1^+^ CD3^+^ T cell number (Mann-Whitney test, *p = 0.047). (**d**) Illustrative flow cytometry plots of contralateral and ipsilateral brain hemispheres show S1P1^+^ T cells in the ischemic tissue.

Given that the kinase SphK1 is a major contributor to S1P generation in several tissues, our results showing upregulation of SphK1 mRNA after ischemia suggested that this condition might increase the cerebral concentration of S1P. Mass spectrometry analysis in the brain tissue 24 h post-ischemia showed elevated S1P levels in the ischemic hemisphere compared with the contralateral hemisphere (Fig. 2a,b). The role of S1P as a T-cell chemoattractant suggests that the ischemia-induced increase in brain S1P concentration is accountable for the higher number of S1P1^+^ T lymphocytes in the ipsilateral versus the contralateral hemisphere, as assessed by flow cytometry (Fig. 2c,d).

### Fingolimod prevents lymphocyte infiltration to the ischemic brain tissue without affecting the infiltration of myeloid cells

**Figure 3:**
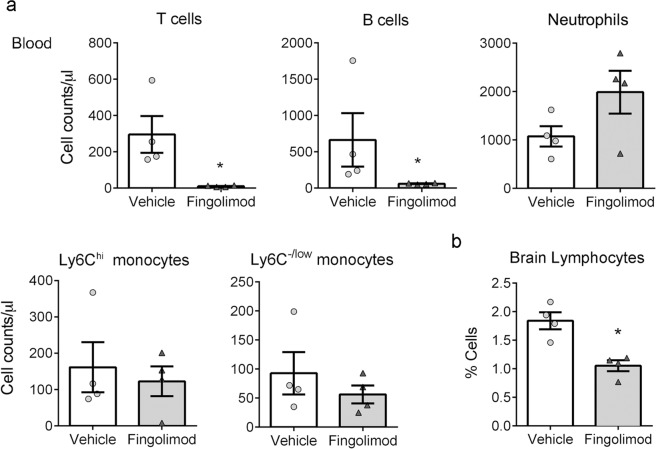
Fingolimod reduces blood lymphocyte counts and lymphocyte infiltration to the ischemic brain. (**a**) Fingolimod induced a strong reduction of T (96%) and B (90%) lymphocytes in the blood 24 h post-ischemia (mean ± SD, n = 4 per group) T cells: 295.8 ± 203.4 cells/μl in the vehicle group and 10.25 ± 4.4 cells/μl in the fingolimod group (Mann-Whitney test, *p = 0.028); B cells: 664.8 ± 737.9 cells/μl in the vehicle group and 61.5 ± 8.7 cells/μl in the fingolimod group (Mann-Whitney test, *p = 0.028). However, it did not modify myeloid cell counts, except for an increase in neutrophils that did not reach statistical significance (Mann-Whitney test, p = 0.200). (**b**) Fingolimod reduced lymphocyte infiltration to the ischemic brain tissue (Mann-Whitney test, *p = 0.029). Bars correspond to the mean ± SEM and symbols show the value of each mouse.

Fingolimod sequesters S1P1^+^ T cells in the lymph nodes preventing them from reaching the inflamed tissues^18,19^. Accordingly, mice treated with fingolimod showed very low lymphocyte counts in the blood (Fig. 3a), and lymphocytes did not increase in the ischemic brain tissue of mice treated with fingolimod, as assessed by flow cytometry 24 h post-ischemia (Fig. 3b).

**Figure 4:**
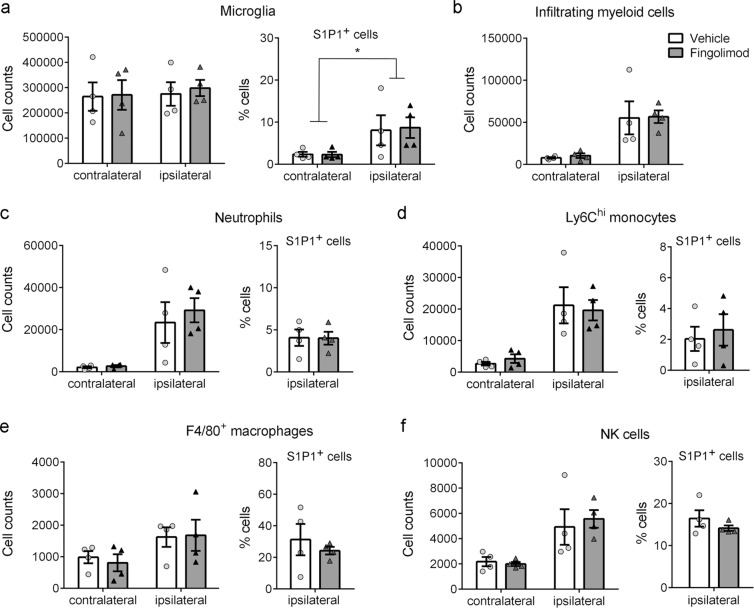
Fingolimod does not affect myeloid and NK cell migration to the ischemic brain tissue. The contralateral and ipsilateral (ischemic) brain hemispheres were analyzed by flow cytometry 24 h post-ischemia (n = 4 mice per group). (**a**) Fingolimod did not affect microglial cell number or microglial S1P1 expression versus the vehicle. However, ischemia increased S1P1 expression in microglial cells regardless of the treatment (two-way Anova, effect of ischemia *p = 0.041). (**b**) Fingolimod did not affect the infiltration of CD45^hi^CD11b^hi^ myeloid cells induced by ischemia. (**c**–**f**) Within the latter infiltrating cells, fingolimod did not modify the numbers and proportion of S1P1^+^ cells in the population of Ly6G^+^ neutrophils (**c**), Ly6C^hi^ monocytes (**d**), and F4/80^+^ macrophages (**e**). (**f**) Likewise, the numbers of NK cells and proportion of S1P1^+^ NK cells was not affected by fingolimod.

Myeloid cells express S1P receptors and migrate along S1P gradients, thus they might be affected by fingolimod. In our experimental model, flow cytometry-based assessment of blood myeloid cell populations revealed no significant differences after fingolimod treatment (Fig. 3a). In the brain, fingolimod did not modify microglial cell number or the proportion of S1P1^+^ microglia versus the vehicle (Fig. 4a). However, we found that ischemia induced S1P1 expression in the microglial cell population (CD45^dim^CD11b^dim^) (Fig. 4a). Importantly, fingolimod did not affect the numbers of myeloid cells (CD45^hi^CD11b^hi^) infiltrating the ischemic tissue (Fig. 4b), including neutrophils (Ly6G^+^) (Fig. 4c), Ly6C^hi^ monocytes (Fig. 4d), and F4/80^+^ macrophages (Fig. 4e). Similarly, NK cells infiltrating the brain were not affected by fingolimod treatment (Fig. 4f). Moreover, fingolimod did not affect the proportion of S1P1^+^ myeloid cells infiltrating the ipsilateral (ischemic) brain hemisphere (Fig. 4c–e). Altogether, these results demonstrate that fingolimod specifically induces T and B cell lymphopenia and prevents the access of lymphocytes to the ischemic brain tissue but does not affect the infiltration of myeloid cells or NK cells. These findings furthermore suggest that S1P receptors are not involved in the transmigration of myeloid cells to the ischemic brain tissue.

### Fingolimod attenuates hemorrhagic transformation independently of lymphocytes

**Figure 5:**
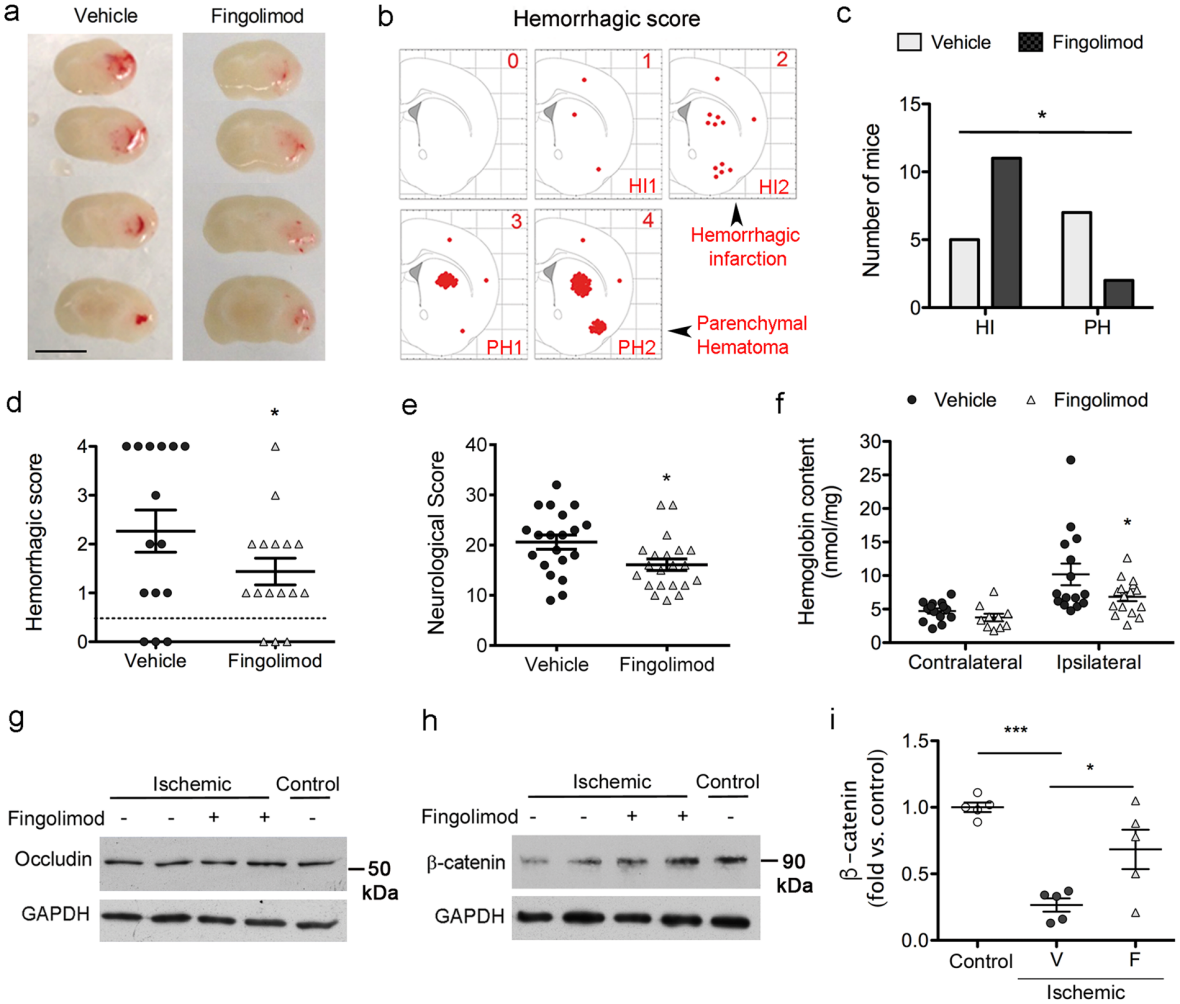
Fingolimod attenuates the degree of hemorrhagic transformation in lymphocyte-deficient mice. Rag2^−/−^ mice were subjected to ischemia and received Fingolimod (1 mg/Kg, i.p.) or vehicle at reperfusion. (**a**) Representative fresh brain tissue sections of Rag2^−/−^ mice treated with Fingolimod (n = 16) or vehicle (n = 15) obtained at 48 h of reperfusion. See expanded data in Supplemental Fig. S1. (**b**) We assigned a hemorrhagic score (HS) following a scale ranging from 0 (no blood in the brain) to 4 (large parenchymal hematoma), intending to compare scores 1 and 2 to hemorrhagic infarction grades HI1 and HI2, and scores 3 and 4 to parenchymal hematoma grades PH1 and PH2, respectively. c-d) Three mice of each treatment group did not show any bleeding. Among the mice with some degree of hemorrhage, PH were less frequent in the fingolimod group (two-sided Chi square, p = 0.025) (**c**), and the mean HS was lower in the fingolimod group (two-sided Mann-Whitney test, p = 0.052) (**d**). (**e**) Fingolimod attenuated the neurological deficit versus the vehicle (n = 20 vehicle group; n = 21 fingolimod group) (Mann-Whitney test, *p = 0.018). f) Brain hemoglobin content in the ipsilateral (ischemic) and contralateral hemispheres showed a small reduction in the ipsilateral hemisphere after fingolimod (n = 16) vs. vehicle (n = 15). Two-way ANOVA by group and hemisphere (*p < 0.05 fingolimod vs. vehicle in the ipsilateral hemisphere). g-h) Representative images of occludin (**g**) and β-catenin (**h**) protein expression determined by western blotting in brain tissue of control and ischemic Rag2^−/−^ mice that received either fingolimod (+) or vehicle (−) (n = 5 per group). Occludin expression was not affected by ischemia or treatment. In contrast, ischemia induced degradation of β-catenin versus control (non-ischemic), and fingolimod (+) attenuated this effect. i) Densitometric analysis of β-catenin western blots showed the group differences (Kruskal-Wallis test followed by Dunn’s test. ***p < 0.001, *p < 0.05). V: vehicle, F: fingolimod.

Lymphocyte-deficient Rag2^−/−^ mice offer a good model to test whether fingolimod has effects independent of lymphocytes. The number of blood lymphocyte counts (mean ± SD), as assessed with a hematology analyzer (ADVIA2, Siemens), was 3.89 ± 1.32 × 10^3^ cells/μl in wild type mice (n = 12) and 0.40 ± 0.19 × 10^3^ cells/μl in Rag2^−/−^ mice (n = 7), which represents a reduction of 90% (Mann-Whitney test, p < 0.001). This reduction is comparable to that induced by treatment with fingolimod in wild type mice (Fig. 3a). We then investigated whether the described protective effect of fingolimod against HT after ischemia/reperfusion^7^ was dependent on lymphocytes. We administered fingolimod or vehicle to lymphocyte-deficient Rag2^−/−^ mice at reperfusion following 45-min MCAo. Two out of 17 mice (12%) in the control group and 4 out of 20 mice (20%) in the fingolimod group died before 48 h. Group differences in the mortality were not statistically significant (Chi-square p = 0.563). We could not obtain the brain of the dead mice due to *rigor mortis* and therefore we excluded these mice from the study. The groups (mean ± SD) did not differ in the following parameters: body weight (26.5 ± 2.5 g vehicle group, and 27.7 ± 3.9 g fingolimod group, Mann-Whitney test p = 0.688), the % drop of cerebral blood flow (CBF) induced by MCAo (85.5 ± 5.5% vehicle group, and 82.4 ± 8.4% fingolimod group, Mann-Whitney test p = 0.295), or the % CBF recovery during the first 15 min after reperfusion in relation to baseline CBF value (98.9 ± 17.0% vehicle group, and 105.6 ± 17.7% fingolimod group, Mann-Whitney test p = 0.329). The degree of bleeding was assessed in fresh brain tissue sections by assigning a hemorrhagic score (HS) ranging from 0 (absence of blood) to 4 (large parenchymal hematoma) (Fig. 5a,b, Supplementary Fig. S1). Factors such as the severity of the lesion induced by the extent of CBF drop during ischemia and the extent and features of CBF recovery at reperfusion, including hyperemia, (Supplementary Fig. S2) might contribute to the variability in the presence and degree of HT after MCAo.

The same number of mice per treatment group showed absence of blood in the brain tissue (HS = 0, n = 3 per group), out of n = 15 mice in the vehicle group and n = 16 mice in the fingolimod group. However, amongst the mice with hemorrhages, intraparenchymal hematomas (HS ≥ 3) were less frequent (Fig. 5c) and the mean hemorrhagic score was lower (Fig. 5d) in the fingolimod group than the vehicle group. Furthermore fingolimod improved the neurological deficit induced by ischemia in Rag2^−/−^ mice (Fig. 5e). Fingolimod also reduced the content of hemoglobin in the ipsilateral hemisphere as measured in brain homogenates of the same mice by a colorimetric assay (Fig. 5f). Altogether, the results show that fingolimod reduced the degree of brain bleeding and neurological deficits but failed to prevent the incidence of HT in lymphocyte-deficient mice.

We next studied the expression of proteins involved in BBB function and vascular integrity in Rag2^−/−^ mice subjected to ischemia and treated with either fingolimod or vehicle, and non-ischemic Rag2^−/−^ mice that were used as controls. Neither ischemia nor fingolimod changed the expression of the tight junction protein occludin (Fig. 5g). However, ischemia caused degradation of β-catenin, a protein of the endothelial adherens junction that is also involved in intracellular signal transduction. Fingolimod attenuated ischemia-induced β-catenin degradation (Fig. 5h,i) supporting some action of the drug on the brain tissue.

### Fingolimod does not reduce brain bleeding under low platelet counts

**Figure 6:**
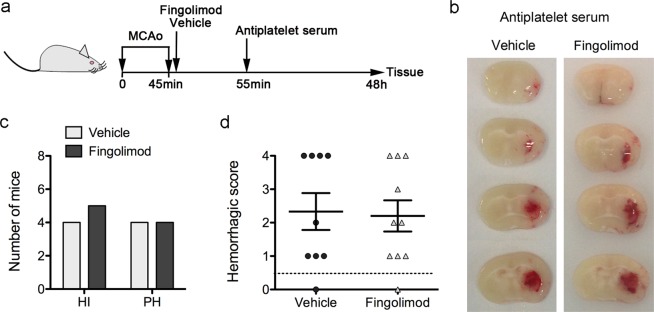
Fingolimod does not reduce hemorrhagic transformation in platelet-depleted mice after severe brain ischemia. (**a**) Schematic illustration of the experimental design. Immediately after reperfusion following 45-min MCAo, wild type mice received an i.p. administration of fingolimod (n = 10) (1 mg/kg) or vehicle (n = 9). Ten minutes later, all the mice received an i.p. administration of anti-platelet serum. At 48 h, fresh brain tissue was obtained to assess for the presence of blood in the ischemic tissue. (**b**) Representative brain sections showing hemorrhages in thrombocypenic mice regardless of whether they received vehicle or fingolimod. (**c**,**d**) A hemorrhagic score (HS) was assigned ranging from 0 (no bleeding) to 4 (large parenchymal hematoma). One mouse per group showed HS = 0. The mice showing some degree of hemorrhage were grouped according to HS categories as follows: HS = 1–2 (hemorrhagic infarction HI1 and HI2) and HS = 3–4 (parenchymal hematomas PH1 and PH2). Two of the mice in the fingolimod group with HS = 4 died at the time of sacrifice (48 h) but the brain was obtained and were included in the analysis. Fingolimod did not reduce the number of platelet-depleted mice with parenchymal hematomas (Chi-square, p = 0.819) (**c**), or the mean HS (Mann-Whitney test p = 0.838) (**d**).

To underscore the potential use of fingolimod to attenuate hemorrhage in conditions at high risk of HT, we investigated whether the drug was able to protect thrombocytopenic mice from HT after ischemia/reperfusion. We used an anti-platelet serum that causes a strong reduction of platelet counts, i.e. platelet counts were undetectable during the first 16 h, and were reduced to 9.7 ± 2.3% and 24.7 ± 15.3% of basal values at 24 h and 48 h, respectively (n = 2 per time point). We depleted platelets in ischemic wild type mice that had received either fingolimod or the vehicle by administering the anti-platelet serum 10 min after reperfusion. Two out of 10 mice of the fingolimod group died during the preparation for sacrifice at 48 h post-ischemia, which allowed brain extraction. Both mice showed large intraparenchymal hematomas (HS = 4), and this information was included in the analysis. None of the 9 mice of the vehicle group died during the time of the study. The groups (mean ± SD) did not differ in body weight (27.9 ± 2.3 g vehicle group, and 28.6 ± 2.8 g fingolimod group, Mann-Whitney test p = 0.556), the % CBF drop during ischemia (80.2 ± 5.6% vehicle group, and 83.4 ± 4.6% fingolimod group, Mann-Whitney test p = 0.200), or the % CBF recovery during the first 15 min after reperfusion (102.4 ± 11.0% vehicle group, and 100.1 ± 14.5% fingolimod group, Mann-Whitney test p = 0.628). Compared to the vehicle, treatment with fingolimod did not reduce the frequency of parenchymal hematomas (HS >3) or the mean HS in platelet-depleted mice (Fig. 6 and Supplementary Fig. S3), indicating that the beneficial effects of fingolimod reducing HT were lost under low platelet counts.

### Fingolimod does not reduce BBB leakage after transient intraluminal MCAo

**Figure 7:**
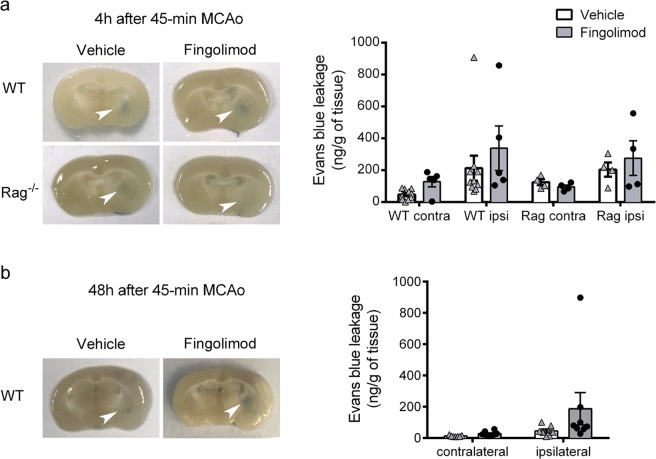
Fingolimod does not prevent ischemia/reperfusion-induced BBB leakage. Evans blue concentration in the ipsilateral (ischemic) and contralateral brain hemispheres. (**a**) 4 h post-ischemia in WT mice treated with fingolimod (n = 5) or vehicle (n = 10) and Rag2^−/−^ mice treated with fingolimod or vehicle (n = 4 per group). Ischemia increased Evans blue extravasation in the ipsilateral hemisphere, but fingolimod did not prevent it (two-way ANOVA by treatment and brain hemisphere; WT mice: hemisphere effect p = 0.026, treatment effect p = 0.196; Rag2^−/−^ mice: hemisphere effect p = 0.055, treatment effect p = 0.749). b) 48 h post-ischemia in WT mice treated with fingolimod (n = 8) or vehicle (n = 7). Again, fingolimod did not reduce Evans blue extravasation (two-way ANOVA; hemisphere effect p = 0.061, treatment effect p = 0.278).

We investigated whether fingolimod could prevent BBB leakage after ischemia/ reperfusion using the Evans blue technique (Fig. 7). Given that BBB breakdown after ischemia is a dynamic phenomenon and shows a peak at around 4 h post-ischemia^29^, we first studied this time point in WT mice and Rag2^−/−^ mice that had received either fingolimod or the vehicle. Differing from our expectation, fingolimod did not prevent ischemia-induced Evans blue extravasation in the ipsilateral brain hemisphere of either genotype (Fig. 7a). We next checked whether fingolimod could prevent BBB leakage at 48 h in WT mice, but again the treatment did not reduce Evans blue extravasation at this time point (Fig. 7b). Together, these findings do not support a role for fingolimod in preventing BBB leakage after transient intraluminal MCAo.

## Discussion

This study reports an upregulation of the S1P pathway in the brain after ischemia/reperfusion followed by attraction of S1P1^+^ lymphocytes to the ischemic brain tissue. Fingolimod-induced lymphopenia prevented lymphocyte infiltration to the injured tissue. Fingolimod reduced the severity of HT but this effect could not be attributed to the actions of the drug on lymphocytes, since it was also detected in lymphocyte-deficient mice. Cerebral ischemia/reperfusion induced a strong, rapid, and transient increase of mRNA expression of the gene encoding for the S1P generating kinase SphK1. The concentration of S1P also increased in the ischemic brain tissue 24 h following ischemia/reperfusion, suggesting a SphK1-mediated effect. The S1P gradient is an important signal for homing of lymphocytes to inflamed tissues^18,19^, that might explain, at least in part, the higher numbers of S1P1^+^ T cells we found in the brain tissue after ischemia/reperfusion. Fingolimod prevents lymphocyte egress from secondary lymphoid organs and access of lymphocytes to the inflamed tissues^18,19^. This effect underlies the action of fingolimod reducing ischemic brain damage^22^, given the deleterious effect of lymphocytes in the acute phase of cerebral ischemia/reperfusion^20,21^. However, fingolimod crosses the BBB and thus may exert direct effects on a variety of brain-resident cells that express S1P receptors^24–28^. The mRNA expression of S1P receptors increased in the brain after ischemia/reperfusion generally starting from 16 h post-ischemia and peaking at approximately day 4. This late peak suggests the possibility that ischemia-induced S1P receptor mRNA increases were related to the cerebral inflammatory response. Among the ischemia-induced enhanced expression of S1P receptor mRNAs, S1P3 mRNA showed the largest increases above control levels. Several lines of evidence suggest that S1P3 mediates the inflammatory responses in astrocytes^30^ and endothelial cells^31,32^. While S1P3 and S1P2 promote inflammation under several experimental conditions, activation of S1P1 is associated with reduction of inflammation^31,32^.

Previous studies showed that fingolimod reduces HT after ischemia/reperfusion^7^. Our results show that this effect is mediated, at least in part, by mechanisms independent of lymphocytes since fingolimod attenuated the extent of bleeding and improved the neurological score in lymphocyte-deficient mice. Accordingly, reduction of the neurological deficits and brain edema in an experimental model of intracerebral hemorrhage mediated by a selective S1P1 agonist in both wild type and Rag2^−/−^ mice^33^ is suggestive of a lymphocyte-independent action. Selective S1P1 drugs may be more powerful against brain hemorrhage than fingolimod given that it provided conflicting results, i.e. fingolimod failed to show protective effects after experimental intracerebral hemorrhage in one of the studies^34^, whereas protective effects were reported in an another study in mice^35^ and in a model of germinal matrix hemorrhage in rat pups^36^. Activation of S1P1 receptor causes cytoskeletal rearrangements promoting barrier functions in the vascular endothelium^27^, and S1P1-deficient mice die before birth due to hemorrhages^37^. We found that ischemia induced degradation of the adherens junction protein β-catenin whereas the expression of the tight junction protein occludin was not affected during the time frame of this study. Disruption of the endothelial adherens junction complex increases paracellular microvascular hyperpermeability in peripheral vascular beds where reduction of β-catenin plays a major role^38^. Furthermore, specific deletion of β-catenin in the endothelium decreases endothelial junctions, causes hemorrhages and is lethal at embryonic stages^39^. Although tight junctions in the brain are critically involved in maintaining BBB function, β-catenin links endothelial adherens junctions with the cytoskeleton and is also essential for the maintenance of BBB integrity. Accordingly, defective β-catenin transcription activity was found to reduce the expression of the tight junction proteins claudin-1 and claudin-3, leading to brain hemorrhage^40^. Furthermore, β-catenin is involved in intracellular signaling of the Wnt pathway^41^ that promotes survival of cerebral microvascular endothelial cells after oxygen-glucose deprivation^42^. In our study, fingolimod attenuated ischemia-induced β-catenin degradation suggesting that it contributed to the preservation of vascular integrity after severe ischemia/reperfusion.

Despite attenuating ischemia-induced HT and β-catenin degradation, fingolimod did not prevent BBB leakage in our experimental model. This finding was unexpected since there is a clear association between increased BBB permeability and HT^43,44^. Nevertheless, HT is a final manifestation of severe vascular damage that progresses through complex steps ranging from initial BBB leakage to vessel rupture and degeneration of the endothelium^4^. Our findings suggest that fingolimod does not prevent ischemia-induced increase in BBB permeability but it might protect from loss of entire vascular integrity. In contrast to our findings, fingolimod reduced Evans blue extravasation in a thromboembolic stroke model where reperfusion was induced by tPA^7^. Given that tPA exacerbates BBB breakdown after brain ischemia^45^, it is plausible that fingolimod prevented damaging effects of tPA on the BBB. Further studies are needed to elucidate the complex effects that fingolimod seems to have on the brain vasculature following ischemic stroke.

Recanalization therapies are not recommended in ischemic stroke patients with low platelet counts (<100,000 counts/μl) due to an increased risk of HT^5^. Platelets were critical players against HT following ischemia/reperfusion as demonstrated by the large hematomas developed by thrombocytopenic mice. Despite promoting barrier function and exerting vasculoprotective effects, fingolimod was unable to reduce bleeding under low platelet counts. Thus, stressing the important hemostatic function of platelets in severe ischemic stroke. Platelet-endothelial interactions might prevent vascular leakage when the endothelium is severely injured^46,47^. Growing evidence supports that platelets can protect the endothelial barrier function by physical coverage of the damaged endothelium or through the local release of soluble vasculoprotective factors, like S1P^48^. However, our results show that fingolimod does not lower the risk or severity of HT after reperfusion following ischemic stroke in mice with low platelets counts.

Altogether, these results show an activation of the S1P signaling pathway in the brain after ischemia/reperfusion and suggests that S1P receptor modulators may play a role in the brain tissue independent of drug effects on the immune system. Fingolimod attenuated HT after cerebral ischemia/reperfusion in a lymphocyte-independent fashion, but the drug was ineffective under thrombocytopenic conditions. The results support protective effects of fingolimod against major vascular disruption despite lack of effect on BBB leakage.

## Methods

### Animals

We used adult (12–16 weeks) male mice (total n = 199). C57BL/6 J wild type mice (#SN 000664, Jaxmice) (n = 127) and lymphocyte-deficient Rag2^−/−^ mice (n = 72) on the C57BL/6 J background (B6(Cg)-Rag2tm1.1Cgn/J, #SN008449, Jaxmice) were obtained from Charles River (Lyon, France). Mice were maintained under controlled SPF conditions in the animal house of the School of Medicine, University of Barcelona. Experiments were conducted with approval of the Ethical Committee of this University, CEEA (Comité Ètic d’Experimentació Animal), according to the Spanish law (Real Decreto 53/2013) and in compliance with the European regulations.

### Brain ischemia

Occlusion of the right middle cerebral artery (MCAo) was carried out under isoflurane anesthesia using a monofilament (#701912PK5Re, Doccol Corporation, Sharon, MA, USA), as described^49^. CBF was monitored with laser Doppler flowmetry (Perimed, AB, Järfälla, Sweden). Reperfusion was induced after 45 min of arterial occlusion. At different time points, the mice were perfused through the heart with heparinized saline under isoflurane anesthesia.

### Neurological score

Two days post-ischemia we assessed the neurological dysfunction with a neurological score modified from a previously reported neuroscore^49^. The score ranged from 0 (no deficits) to 37 (poorest performance in all items) and it is calculated as the sum of the general and focal deficits, including the following general deficits (scores): hair (0–2), ears (0–2), eyes (0–3), posture (0–3), spontaneous activity (0–3), and epileptic behavior (0–1); and the following focal deficits: body symmetry (0–2), gait (0–4), climbing (0–3), circling behavior (0–3), forelimb symmetry (0–4), compulsory circling (0–3), and whisker response (0–4).

### Drug treatment

Treatment was randomly allocated using a random list that assigned a given treatment (A/B) to each mouse, and treatment was administered in a blinded fashion. Fingolimod (1 mg/kg) (#10006292, Cayman Chemical, Ann Arbor, MI, USA) or the vehicle (saline containing 0.5% DMSO) was administered i.p. immediately after reperfusion following MCAo. For platelet depletion, mice received an i.p. injection of rabbit anti-mouse platelet serum (#CLA31440, Cedarlane, Burlington, NC, USA) (15 μl diluted in 200 μl of sterile saline) 10 min after reperfusion.

### Histological assessment of hemorrhagic transformation

The brain was cut in 1mm-thick sections and pictures of fresh tissue were obtained to assess HT. A semi-quantitative method was used to evaluate the severity of brain hemorrhage by giving scores (HS) from 0 to 4, where 0 corresponds to absence of hemorrhage, 1–2 correspond to increasing grades of intracerebral hemorrhage (IH1 and IH2), and 3–4 correspond to parenchymal hematomas (PH1 and PH2) (see Fig. 5b).

### Quantification of brain hemoglobin content

Mice were anesthetized and perfused through the heart with a phosphate-buffered saline pH 7.4, containing 0.16 mg/mL heparin. The brain was sectioned to obtain the hemorrhagic score as above. The ipsilateral (ischemic) and contralateral hemispheres were separated in each sections and the tissue of each hemisphere was pulled and processed to measure the brain hemoglobin content with a colorimetric assay (#700540, Cayman Chemical).

### qRT-PCR

The ipsilateral and contralateral brain hemispheres were dissected out, frozen, and kept at −80 °C. RNA was extracted from brain tissue (Purelink RNA Kit, Invitrogen) and total RNA (500–1,000 ng) was reverse-transcribed using a mixture of random primers (High Capacity cDNA Reverse Transcription kit, Applied Biosystems, Foster City, CA, USA). Quantitative real-time RT-PCR analysis was carried out by the Taqman system (#4304437, Life Technology, Carlsbad, CA, USA) using the iCycler iQTM Multicolor Real-Time Detection System (Bio-Rad, Hercules, CA, USA).

### Measurement of S1P concentration by mass spectrometry

To extract S1P from the brain tissue, the contralateral and ipsilateral brain hemispheres were separated, weighed and homogenized in 1 mL of 1 M NaCl. S1P labeled with 7 deuterium (D-erythro-sphingosine-d7-1-phosphate, Avanti Polar Lipids, Alabaster, Alabama, USA) (30 μM) was added as the internal standard. After mixing the sample with 200 μl 6 M HCl, samples were mixed with 2 mL CHCl_3_. Samples were centrifuged at 19,000 xg for 3 min. The lower organic phase was transferred into a glass centrifuge tube using a glass Pasteur pipette and CHCl_3_ was evaporated in a vacuum concentrator. Samples were dissolved in 100 μl MeOH:CHCl_3_ (4:1) and analyzed by LC-MS/MS on an API 3000 (AB Sciex) triple quadrupole mass spectrometer integrated on an Acquity UPLC system (Waters Corporation, Milford, MA, USA). A Kinetex (Phenomenex, Torrance, CA, USA) 2.6 µm XB C18 100 Å 50 × 2.00 mm column was used as stationary phase. The column temperature was set at 60 °C. The Mobile phases consisted of (A) 75% methanol, 0.1% formic acid, and 5 mM ammonium formiate, and (B) 100% methanol, 0.1% formic acid, and 5 mM ammonium formiate. The gradient elution profile was as follows: 0 min 100% A, from min 0 to min 2 the % A decreased from 100% to 0%, and was kept at 0% until min 5.5, when it was returned to initial conditions at minute 5.6 until minute 6. Flow was set at 0.6 mL/min. Analyte was detected in positive electrospray ionization (ESI) mode. The ion spray voltage was set at 5000 Volts and source temperature at 400 °C. Data was acquired in multiple reaction monitoring (MRM) mode. Transitions selected were 380.1/264.4 for S1P and 387.3/271.5 for the internal standard. Results were integrated and calculated using linear regression by the Analyst 1.4.2 software (AB Sciex).

### Flow cytometry

Cell isolation from brain tissue was performed as described^50^. Cells were incubated at 4 °C for 10 min with FcBlock followed by primary antibodies anti-CD3 (#557869; clone 17A2; Alexa Fluor-647; BD Pharmingen, San Diego, CA, USA), anti-CD45 (#553080; clone 30-F11;FITC, Horizon V500; BD Pharmingen,), anti-CD11b (#557657; clone M1-70; APC-Cy7; BD Pharmingen) and anti-S1P receptor-1 (S1P1) (#FAB7089P; clone 713412; PE; R&D Systems, Minneapolis, MN, USA). Brain cells were studied in a BD FacsCanto II cytometer using the FacsDiva software (BD Biosciences, San Jose, CA, USA). Another set of flow cytometry data were acquired in a BD LSRII cytometer after staining blood and brain cells with cell/dead fixable aqua dead stain (#L34957; Molecular Probes, Eugene, OR, USA), anti-CD45R (#560472; clone RA3–6B2; V450; BD Biosciences), anti-F4–80 (#123133; clone BM8; Brilliant Violet-605; Biolegend, San Diego, CA, USA), anti-CD45 (#564225; clone 30-F11; Brilliant Violet-786; BD Horizon), anti-Ly6C (#AB15686; clone ER-MP20; FITC; Abcam, Cambridge, UK), anti-CD161(#65-5941; clone PK136 PerCP/Cy5.5; Tonbo Biosciences, San Diego, CA, USA), anti-S1P1 PE, anti-γδ TCR (#25-5711-82; clone GL3; PE-Cy7; eBioscience, San Diego, CA, USA), anti-CD3 Alexa Fluor-647, anti-Ly6G (#127622; clone 1A8; Alexa Fluor-700; Biolegend), and anti-CD11b APC-Cy7. Data analysis was performed with FlowJo software (v10 FlowJo, LLC, Ashland, OR, USA). Cell count was determined by addition of fluorescent beads (#7547053; Beckman Coulter, Brea, CA, USA) during acquisition.

### Western blotting

For Western blotting, we obtained tissue from the ipsilateral (ischemic) and contralateral brain hemispheres. Proteins were extracted in radio-immunoprecipitation buffer (RIPA) containing a protease inhibitor cocktail (cOmplete^TM^, Roche, Basel, Switzerland). Electrophoresis was carried out in 10% polyacrilamide gels and proteins were transferred to membranes (Immobilon-P, PVDF, #IPVH00010, MilliporeSigma, Merck, Burlington, MA, USA) that were incubated overnight at 4 °C with one of the following antibodies: a mouse monoclonal antibody anti-β-catenin (#610154, BD Biosciences) diluted 1:16,000, or a rabbit polyclonal antibody anti-occludin (#71–1500, Invitrogen, ThermoFisher Scientific, Waltham, MA, USA) diluted 1:250. A rabbit polyclonal antibody anti-GAPDH (#ab181602, Abcam, Cambridge, UK) diluted 1:20,000, or a mouse-monoclonal antibody anti-β-tubulin (#T4026, Sigma) diluted 1:500,000 were used as the loading controls. Quantification of band intensity was carried out with Quantity One 1-D analysis software (Bio-Rad, Hercules, CA, USA). The ratio of the signal intensity versus the loading control was calculated. Values are expressed as fold vs. the mean value of control tissue run in each blot.

### Blood-brain barrier permeability

We studied Evans blue extravasation to the brain tissue as reported with minor modifications^51^. In brief, a solution of 2% Evans blue (Sigma-Aldrich) (w/v in saline) was administered i.v. (4 μl/g of body weight) 3 h or 47 h after reperfusion. One-hour later the animals were anesthetized and perfused through the heart with saline. The brain tissue was removed from the skull, and the ipsilateral and contralateral hemispheres were separated and were independently processed. Samples were weighed and immersed in a solution of 50% trichloroacetic acid (2 mL/g of tissue), homogenized, and centrifuged 12,000 × g for 20 min at 4 °C. The supernatant was diluted 1:3 in 100% ethanol, and the content of Evans blue was measured in a fluorimeter at 680 nm. We also obtained blood samples to ensure that the plasmatic Evans blue concentration was similar between mice. The concentration of Evans Blue was calculated by using a standard curve generated with known concentrations of the dye.

### Statistical analyses

Two-group comparisons were carried out with the Mann–Whitney U test. Multiple groups were compared with the Kruskal-Wallis test followed by subsequent post-hoc analysis with the Dunn’s test. Two-way ANOVA followed by the Bonferroni test was used to compare treatment groups by brain hemisphere. Chi-square was used to compare frequencies between groups. Statistical analyses were performed using GraphPad v5 software (GraphPad Software Inc., La Jolla, CA,USA). The specific tests and n values for each experiment are reported in each figure legend.

## Supplementary information

Supplementary data

## Data Availability

Most data is provided in the manuscript and Supplementary Information. Other data will be available from the corresponding author upon reasonable request.
