# Supplementary material for: Role of the S1P pathway and inhibition by fingolimod in preventing hemorrhagic transformation after stroke
Source: Sci Rep. 2019 Jun 5;9:8309. doi: 10.1038/s41598-019-44845-5 (PMC6549179; doi:10.1038/s41598-019-44845-5)

SUPPLEMENTARY FILE

Role of the S1P pathway and inhibition by fingolimod in preventing hemorrhagic transformation after stroke

Angélica Salas-Pérdomo[1,2], Francesc Miró-Mur[2], Mattia Gallizioli[1,2], Vanessa H. Brait[2,#], Carles Justicia[1,2], Anja Meissner[2], Xabier Urra[2,3], Angel Chamorro[2,3], Anna M. Planas[1,2,*]

[1] Departament d'Isquèmia Cerebral i Neurodegeneració, Institut d'Investigacions Biomèdiques de Barcelona (IIBB), Consejo Superior de Investigaciones Científicas (CSIC), Barcelona, Spain

[2] Àrea de Neurociències, Institut d'Investigacions Biomèdiques August Pi i Sunyer (IDIBAPS), Barcelona, Spain

[3] Functional Unit of Cerebrovascular Diseases, Hospital Clínic, Barcelona, Spain

[#] Current address: The Florey Institute of Neuroscience and Mental Health, University of Melbourne, Parkville, Victoria, Australia.

* Corresponding author:

Anna M. Planas
IIBB-CSIC, IDIBAPS
Rosselló 161, planta 6
08036-Barcelona, Spain
e-mail: anna.planas@iibb.csic.es
Tel: +34-93 363 83 27
Fax: +34-93 363 83 01

Supplementary Figures

Supplementary Fig. S1

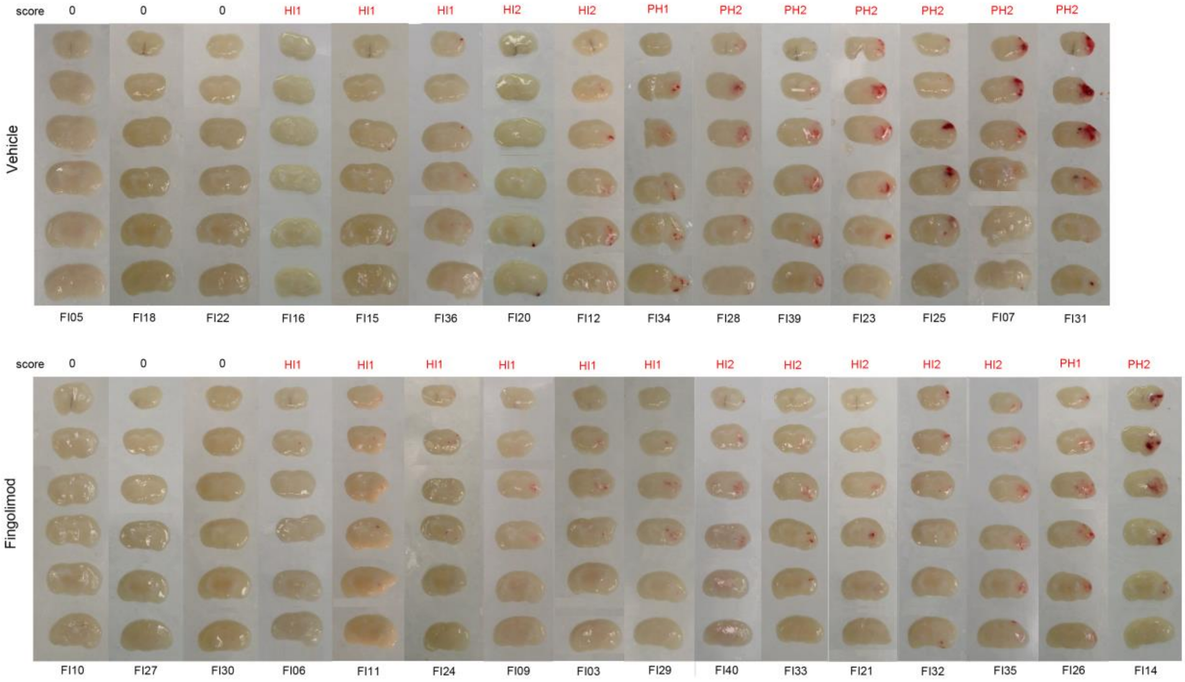

Supplementary Figure S1. Fingolimod attenuates hemorrhagic transformation in Rag2$^{-/-}$ mice after 45-min MCAo and 48h reperfusion. Rag2$^{-/-}$ mice received an i.p. administration of Fingolimod (n=16) (1mg/kg) or vehicle (n=15) after reperfusion following 45-min MCAo. At 48h mice were perfused with saline and fresh brain tissue 1mm-thick slices were obtained to assess the presence of blood in the infarcted brain tissue. In addition to these mice, two mice of the vehicle group and 4 mice of the fingolimod group died before 48h and the brain could not be recovered. A hemorrhagic score was assigned ranging from 0 (no bleeding) to 4 (large parenchymal hematoma), intending to compare scores 1 and 2 to hemorrhagic infarction grades HI1 and HI2, and scores 3 and 4 to parenchymal hematoma grades PH1 and PH2, respectively.

Supplementary Fig. S2

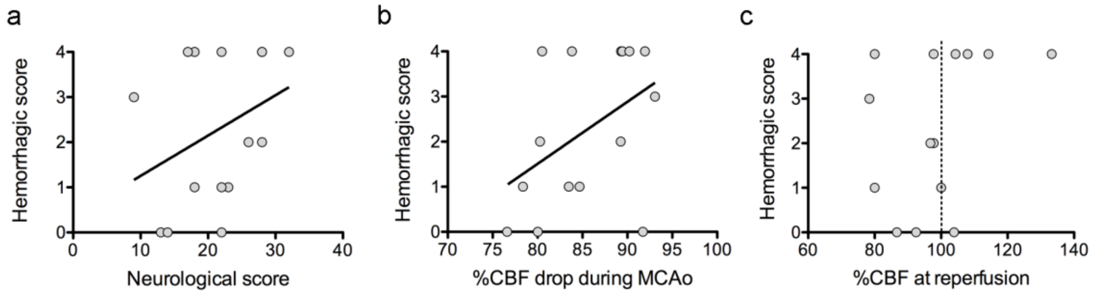

Supplementary Figure S2. Relationship between the hemorrhagic score and the neurological score and CBF data. As an exploratory study to find out possible contributors to HT, we examined the relationship between the hemorrhagic score (ranging from 0 in the absence of HT to 4 for large parenchymal hematomas) and several parameters obtained in each mice (Rag2$^{-/-}$ mice treated with the vehicle (n=15) and presented in Fig. 5). The hemorrhagic score showed some tendency to relate to the neurological score (a) but the correlation was not statistically significant (Spearman r=0.389, p=0.152). The relationship between the % drop in CBF during MCAo and the hemorrhagic score (b) showed a trend towards statistical significance (Spearman r=0.461, two-tailed p=0.083). Finally, regarding the % CBF at reperfusion (values correspond to the mean %CBF value obtained during the first 15 min after reperfusion) (c), we observed that mice showing hyperemia in relation to the baseline CBF value seemed to be prone to develop HT.

Supplementary Fig. S3

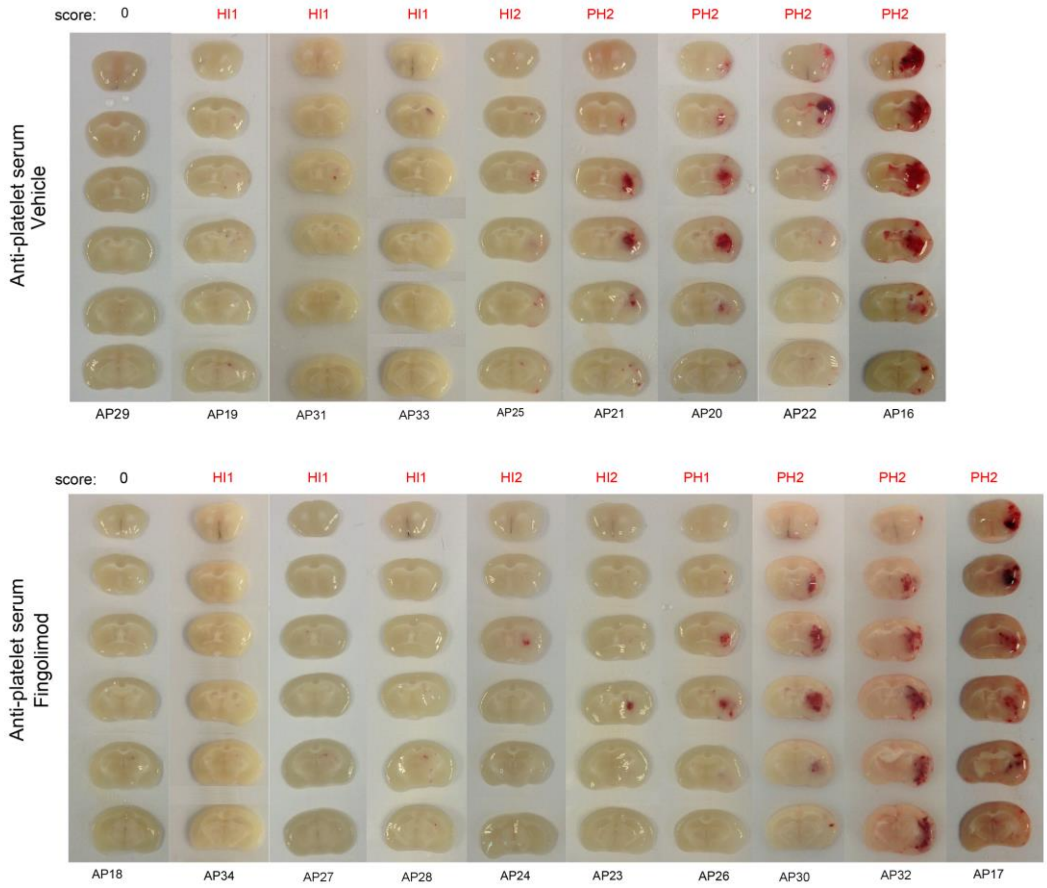

Supplementary Figure S3. Fingolimod does not prevent hemorrhagic transformation in platelet-depleted mice after severe brain ischemia. Immediately after reperfusion following 45-min MCAo, wild type mice received an i.p. administration of fingolimod (n=10) (1mg/Kg) or vehicle (n=9). Ten min later, all the mice received an i.p. administration of anti-platelet serum. At 48h mice were perfused with saline and 1mm-thick slices of fresh brain tissue were obtained to assess the presence of blood in the infarcted tissue. The two mice on the right-hand side of the fingolimod group, with HS=5 (codes AP32 and AP17), died immediately before the programmed time of sacrifice at 48h (AP32 could still be perfused through the heart with saline but AP17 was not perfused). None of the mice in the vehicle group died before 48h. A hemorrhagic score was assigned ranging from 0 (no bleeding) to 4 (large parenchymal hematoma), intending to compare scores 1 and 2 to hemorrhagic infarction grades HI1 and HI2, and scores 3 and 4 to parenchymal hematoma grades PH1 and PH2, respectively.

ORIGINAL IMAGES OF WESTERN BLOTS SHOWN IN FIGURE 3

Lanes shown in the blots are the last 5 lanes on the right hand side.

MEMBRANE β-catenin

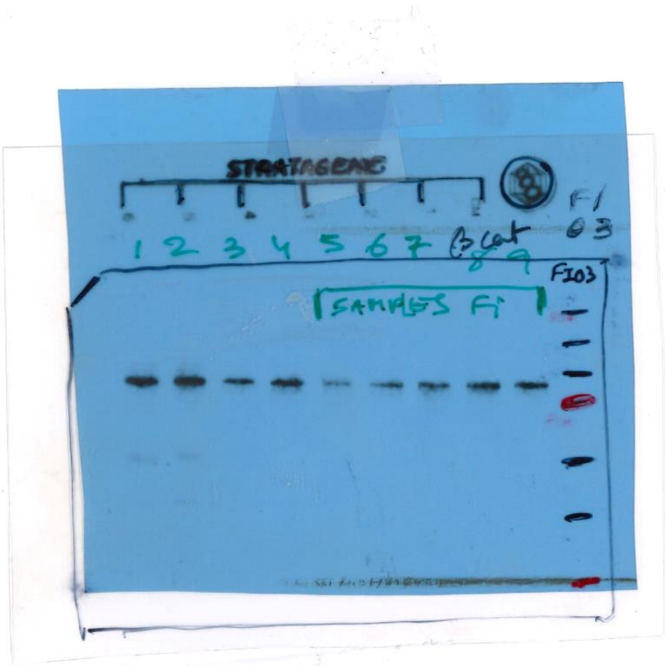

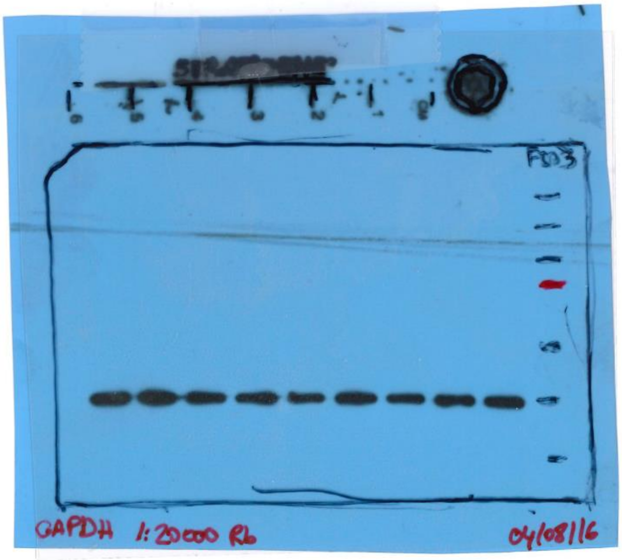

MEMBRANE OCCLUDIN

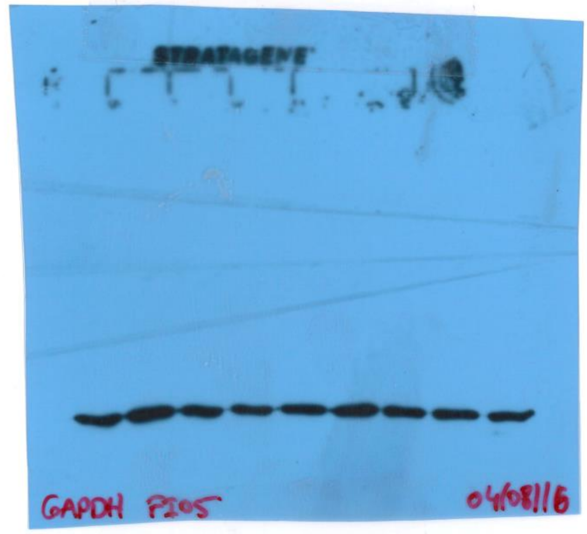

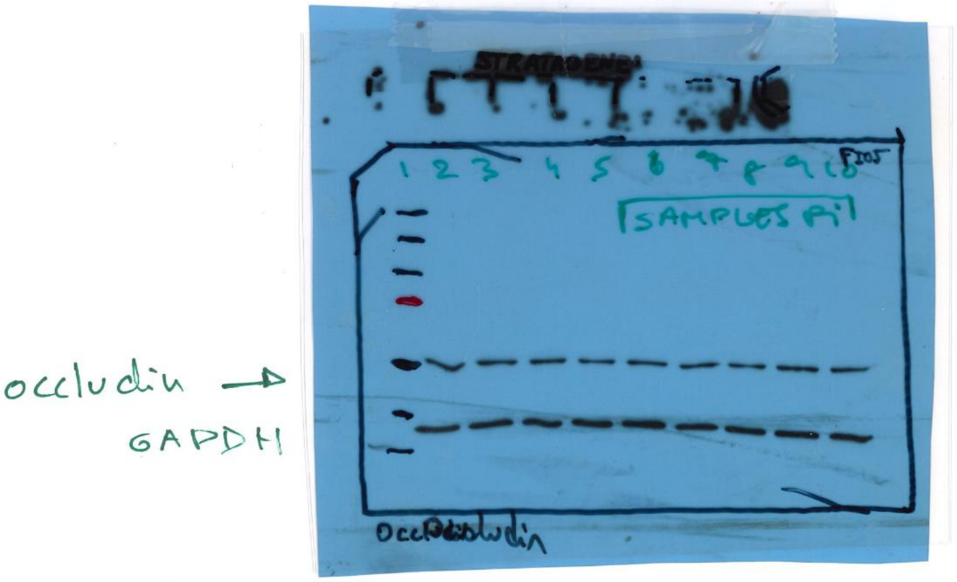

Supplement: Supplementary file 1 — Supplementary data [file 41598_2019_44845_MOESM1_ESM.pdf]
